# The tetraspanin CD151-ARSA mutant inhibits angiogenesis via the YRSL sequence

**DOI:** 10.3892/mmr.2012.1250

**Published:** 2012-12-24

**Authors:** DAN PENG, HOUJUAN ZUO, ZHENGXIANG LIU, JIN QIN, YUANLIN ZHOU, PENGCHENG LI, DAOWEN WANG, HESONG ZENG, XIN A. ZHANG

**Affiliations:** 1 Department of Nuclear Medicine, Tongji Medical College, Huazhong University of Science and Technology, Wuhan 430030, P.R.China; 2 Department of Cardiology of Tongji Hospital, Tongji Medical College, Huazhong University of Science and Technology, Wuhan 430030, P.R.China; 3 Department of Physiology and Cancer Center, University of Oklahoma Health Science Center, Oklahoma City, OK, USA

**Keywords:** CD151, vesicular trafficking, human umbilical vein endothelial cells, angiogenesis

## Abstract

Previous studies have shown that the tetraspanin CD151 is essential for pathological or physiological angiogenesis. However, the cellular signaling mechanism and the role of the CD151 YRSL sorting motif in *in vitro* vasculogenesis remains unknown. In this study, the results showed that both CD151 and CD151-ARSA gene delivery were capable of increasing the expression of CD151 at the protein level in human umbilical vein endothelial cells (HUVECs). Moreover, there was no significant difference in CD151 protein expression between the CD151 group and the CD151-ARSA group. Overexpression of CD151 promoted HUVEC cell proliferation, migration and capillary network formation *in vitro*. However, in the CD151-ARSA group, the abilities of cell proliferation, migration and capillary network formation were all decreased, compared with the CD151 group. Furthermore, the activation of PI3K, Akt and ERK signaling pathways was attenuated in the CD151-ARSA mutant group compared with the CD151 group. This study suggests that the YRSL motif of CD151 plays a key role in CD151-induced angiogenesis. Our observations provide insights into a new mechanism of CD151 regulating angiogenesis via vesicle trafficking.

## Introduction

CD151, a tetraspanin superfamily protein, contains two extracellular loops, four hydrophobic transmembrane domains and two short cytoplasmic tails (1,2). This tetraspanin is expressed broadly in various tissues and is particularly abundant in endothelia, epithelia, smooth muscle and megakaryocytes (3,4). At the cellular level, CD151 is characteristically localized in intracellular vesicles and at cell-cell junctions in endothelial cells (ECs) (4). Moreover, the association of CD151 with integrins stands out as a prominent feature (3,5,6). A ‘CD151-integrin’ complex model has been proposed and this model is functionally linked to CD151-induced biological processes (6–9).

Previous studies have shown that CD151 is involved in regulating cell motility, cell-cell adhesion and contact, tumor metastasis and invasion. At present, accumulating evidence has revealed a role of CD151 in angiogenesis (4–6). Furthermore, the regulatory role of CD151 in angiogenesis was supported by CD151 knockout studies (10,11). Our group focuses on the role of CD151 in angiogenesis: we previously demonstrated that delivery of the CD151 gene was capable of increasing angiogenesis in a pig myocardial ischemia model and a rat ischemic hindlimb model (12,13). CD151 transfection enhanced ECs proliferation, migration and capillary network formation on Matrigel (14,15). Therefore, CD151 may promote angiogenesis *in vivo* and *in vitro*. Although the activation of PI3K and ERK signaling pathways was demonstrated to be involved in CD151-induced angiogenesis (15–17), the mechanisms involved remain unclear.

As previously shown, vesicle trafficking is a fundamental membrane trafficking event in cell migration, and endocytic trafficking pathways have become established in recent years as being important in the regulation of intracellular signaling pathways during processes such as cell division, migration and angiogenesis (18–21). Recently, several studies noted that vesicle trafficking was also one characteristic feature of CD151 and observed that the YRSL sequence of CD151 was necessary for internalization and vesicle trafficking of CD151 (22). It was revealed that the C-terminal cytoplasmic domain of CD151 contains a YRSL sequence or YXXϕ sorting motif, in which Y is tyrosine, X is any amino acid, and ϕ represents the amino acid residue with a bulky hydrophobic side chain (23,24). Mutation of this CD151 YRSL motif markedly attenuated internalization and vesicle trafficking of CD151 (22). The YRSL motif-mediated internalization of CD151 was thought to promote cell migration by modulating the endocytosis and/or vesicular trafficking of CD151 (22). Hence, we hypothesized that the YRSL motif of CD151 may be responsible for the regulation of CD151-related human umbilical vein endothelial cell (HUVEC) migration and capillary network formation *in vitro*, and CD151 may affect angiogenesis via vesicle trafficking.

In this study, we mutated CD151 YRSL→ARSA, which was capable of impairing the vesicle trafficking of CD151 (22). We then examined the roles of CD151 and the CD151-ARSA mutant in the cell proliferation, migration and capillary network formation of HUVECs, with a recombinant adeno-associated virus (rAAV) construct encoding CD151 and the CD151-ARSA mutant. The purpose of the present study was to investigate the critical role of the YRSL sequence of CD151 during angiogenesis *in vitro* and the mechanism(s) involved.

## Materials and methods

### Materials and reagents

All cell culture reagents were obtained from Gibco BRL Life Technologies, Inc. (Grand Island, NY, USA), including Dulbecco’s modified Eagle’s medium (DMEM/F12), trypsin and fetal bovine serum (FBS). Apigenin was supplied by Calbiochem-Novabiochem (Darmstadt, Germany). Rabbit anti-ERK1 and anti-phospho-ERK1 antibodies were purchased from New England Biolabs (Beverly, MA, USA). The restriction enzymes were purchased from Takara (Dalian, China). Antibodies against PI3K (P110), Akt, phospho-Akt (ser473), CD151 and β-actin were purchased from Santa Cruz Biotechnology Inc. (Santa Cruz, CA, USA). LY294002 and Hybrisol solution were purchased from Intergen (Purchase, NY, USA). Tween-20, PMSF and aprotinin were purchased from B&D Biosciences (Heidelberg, Germany). All other chemicals and reagents were purchased from Sigma-Aldrich Inc. (Shanghai, China) unless otherwise specified. This study was approved by the ethics committee of HuaZhong University of Science and Technology.

### Construction of pAAV-CD151, pAAV-CD151-ARSA and pAAV-GFP

The PzeoSV-CD151 plasmid was described in an earlier study (6). The construction of pAAV-CD151, pAAV-anti-CD151 and pAAV-green fluorescent protein (GFP) has been described previously (12–15). The CD151-ARSA mutant was generated by recombinant PCR. The pAAV-CD151 vector contained the full-length wild-type human CD151, and CD151 was used as the template. For the CD151-ARSA mutant, the following primers were used: ATGATCTTCACGTGCT GCCTGGCTAGGAGTGCCAAGCTGGAGCACTACGCCT ACCCC (internal sense primer to amplify the 3′-region) and GGGGTAGGCGTAGTGCTCCAGCTTGGCACTCCTAGC CAGGCAGCACGTGAGATCAT (internal antisense primer to amplify the 5′-region) of the CD151 template. We then used either external sense GCTTAGATCTGCCACCATGGGTGA GTTCAACGAG or external antisense GACGCGGCCGCT CAGGCGTAGTCGGG primers. The final recombinant PCR was performed using purified PCR products and external sense and antisense primers. The final PCR products were incised by the *Bgl*II and *Not* restriction enzymes, and then the incised products were purified and ligated into the adeno-associated virus (AAV) vector at the *Bam*HI and *Not*I restriction sites. Proper ligation was confirmed by sequencing.

### Preparation of recombinant adeno-associated viruses (rAAVs)

The rAAV vector pXXUF1, packaging plasmid pXX2, adenovirus helper plasmid pHelper, and a rAAV plasmid containing GFP cDNA was obtained from Dr Xiao Xiao (University of North Carolina, Chapel Hill, NC, USA). The packing and production of rAAV-GFP, rAAV-CD151 and rAAV-CD151-ARSA were carried out using a triple-plasmid cotransfection method in human embryonic kidney cells [293 cells, American Type Culture Collection (ATCC), Manassas, VA, USA] (12,13). For purification, a single-step gravity-flow column was applied (25). The titers of vector particles were determined.

### HUVEC culture and transfection

HUVECs were obtained from ATCC and grown in DMEM/F12 medium supplemented with 10% FBS (Gibco), streptomycin 100 μg/ml and penicillin 100 U/ml (all obtained from Sigma) at 37°C under 5% CO_2_ and 95% air. Only cells passaged less than five times were used for experiments. Cells were grown to 50–60% confluence and transfected with rAAV-GFP, rAAV-CD151 and rAAV-CD151-ARSA, as described previously (14). For the control group, PBS or HD-Fugene was added. Cells were incubated in the conditions above for 3 days and then subjected to the following assays.

### Cell proliferation assay

Assessment of cell viability was performed using the Cell Counting Kit-8 (CCK-8) assay. At 12, 24 and 48 h after rAAV transfection, HUVECs were incubated in 10% CCK-8 (Beyotime Institute of Biotechnology, Nantong, China) diluted in normal culture medium at 37°C until visual color conversion occurred in 96-well culture plates. The number of viable cells was assessed by measurement of absorbance at 450 nm using a microplate reader.

### Cell migration assay

Assessment of cell migration was performed using a cell wound-healing assay. Briefly, transfectant cells were cultured in 60-mm diameter dishes and synchronized in 0.5% FBS. After HUVECs were grown to confluence, wounds were generated by scraping the monolayers with sterile pipette tips. After 0, 12, 24 and 48 h culture at 37°C, respectively, images of the wound in each well were captured using an inverted microscope (Nikon TE 2000; Nikon, Tokyo, Japan).

### Capillary network formation assay

Assessment of cell migration was performed using capillary network formation on Matrigel. Briefly, Matrigel (0.5 ml) was polymerized on 24-well plates, and 5×10^4^ transfectant cells were then plated in full-growth medium for 1 h. Once the cells were seeded, the medium was replaced with medium containing 0.5% serum. After incubation at 37°C for 12, 24 and 48 h, the capillary network formation was visualized using an inverted microscope (Nikon TE 2000) equipped with digital imaging. For each treatment, 10 field images were captured, and the area containing endothelial tubes and networks that had formed was quantified using the Scion Image Analysis System (Windows version of Scion Image, NIH) with background subtraction.

### Protein extraction and western blotting

HUVEC proteins were extracted and used for western blot analysis (12,14), with specific primary antibodies against CD151, PI3K, Akt, phospho-Akt, ERK1, phospho-ERK1 and β-actin. The HRP-conjugated secondary antibodies were used respectively to reveal the specific protein bands with ECL detection reagents. The intensities of protein bands were quantified by densitometry. In addition, inhibitors of MAPK (apigenin) and PI3-kinase (LY294002) were added to cultured HUVECs. The protein levels of PI3K, Akt, phospho-Akt, ERK1 and phospho-ERK1 were observed.

### Statistical analysis

Data were analyzed using SPSS 18.0 statistical software (Chicago, IL, USA). Data were presented as the means ± standard deviation (SD) unless otherwise specified. Statistical comparisons between two groups were carried out using the Student’s t-test or one-way ANOVA. P<0.05 was considered to indicate a statistically significant result.

## Results

### Expression of CD151 protein after transfection

**Figure 1:**
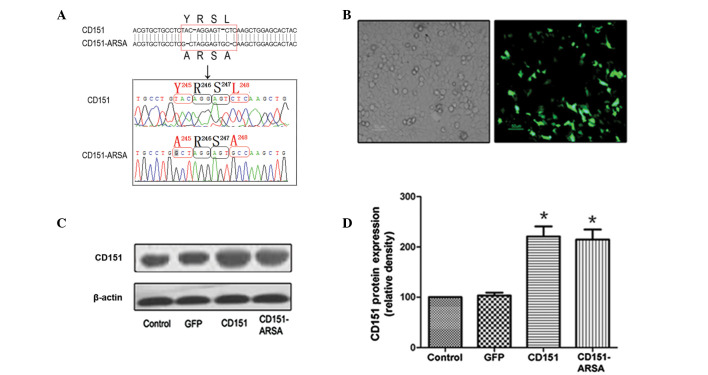
Expression of CD151 protein after transfection in HUVECs. (A) The gene sequences of CD151 and CD151-ARSA. The CD151 mutation (CD151-ARSA) changed the motif of YRSL245-248 to ARSA245-248. (B) HUVECs transfected with rAAV-GFP observed using an inverted fluorescence microscope 7 days after transfection (the same field). (C) Western blot analysis. (D) Quantitative analysis of CD151 protein expression. β-actin was used as an internal loading control. The mean density of CD151 in control group was defined as 100%. Each experiment was performed at least in triplicate. ^*^p<0.05 vs. control and GFP group. HUVECs, human umbilical vein endothelial cells; rAAV, recombinant adeno-associated virus; GFP, green fluorescent protein.

In the present study, we mutated the YRSL motif in the human CD151 molecule. Gene sequencing analysis showed that the Y and L residues in the CD151 YRSL motif were replaced with alanine (A) residues simultaneously, and the resulting **Y**RS**L**→**A**RS**A** mutant was designated as the CD151-ARSA mutant (Fig. 1A). As shown in Fig. 1B, HUVECs transfected with rAAV-GFP were observed using an inverted fluorescence microscope.

Compared with the control group and the GFP group, the expression of CD151 protein was increased significantly in the CD151 and CD151-ARSA groups (Fig. 1C and D). However, there was no significant difference in CD151 protein expression between the CD151 group and the CD151-ARSA mutant transfectant group (Fig. 1C and D). The experiment suggests that the CD151-ARSA mutant does not affect the expression of CD151 protein.

### Effects of CD151 and CD151-ARSA transfection on the proliferation of HUVECs

**Figure 2:**
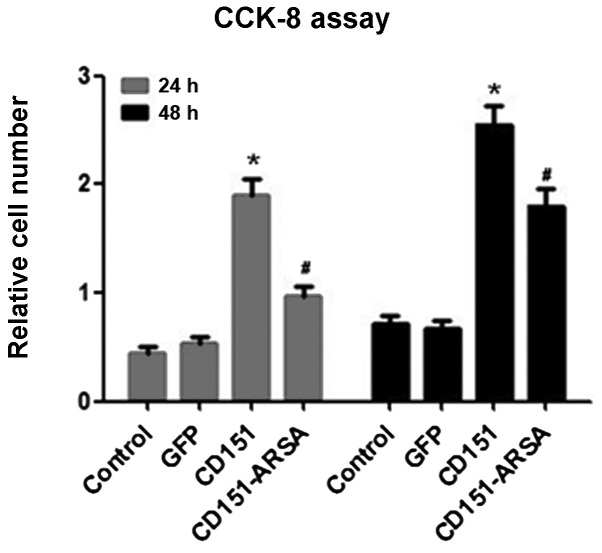
Effects of CD151 and CD151-ARSA transfection on HUVECs. Cell Counting Kit-8 (CCK-8) assays were performed at 24 and 48 h after gene delivery. The CD151 group showed promoted proliferation ability. In the CD151-ARSA mutation group, the proliferation of HUVECs was decreased significantly at 24 or 48 h after delivery. Three independent experiments were carried out, and each experiment was in triplicate. ^*^p<0.05 vs. control and GFP group. ^#^p<0.05 vs. CD151 group. HUVECs, human umbilical vein endothelial cells; GFP, green fluorescent protein.

To determine the proliferative effects of CD151 and CD151-ARSA, we performed the CCK-8 assay at 12, 24 and 48 h after delivery. As shown in Fig. 2, the CD151 group showed increased proliferation ability, compared with the control group and the GFP group at 24 and 48 h after rAAV transfection. No significant difference was observed in the groups at 12 h after transfection (data not shown). However, the results showed that the CD151-induced proliferation of HUVECs was decreased significantly by the CD151-ARSA transfection, at 24 or 48 h after delivery (Fig. 2). These data suggest that the CD151-ARSA mutant was capable of impairing the cell proliferation ability of CD151.

### Effects of CD151 and CD151-ARSA transfection on the migration of HUVECs

**Figure 3:**
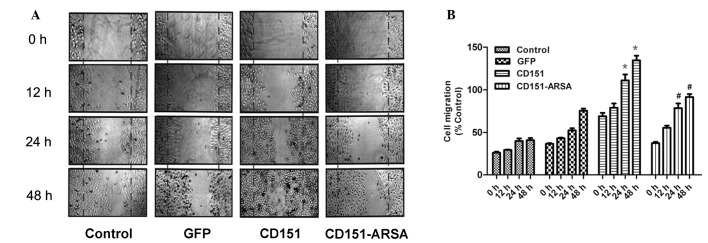
Effects of CD151 and CD151-ARSA transfection on the migration of HUVECs. (A) Cell migration was assessed by a cell wound-healing assay and observed at 0, 12, 24 and 48 h after rAAV transfection. (B) Quantitative analysis of HUVEC migration. Each experiment was performed at least in triplicate. ^*^p<0.05 vs. control and GFP group. ^#^p<0.05 vs. CD151 group. HUVECs, human umbilical vein endothelial cells; GFP, green fluorescent protein.

Regulation of cell motility is a prominent feature of CD151 (4,22). Cell migration was assessed by a cell wound-healing assay and observed at 0, 12, 24 and 48 h after CD151 transfection. As shown in Fig. 3A and B, the expression of CD151 significantly enhanced cell migration at 24 and 48 h, compared with the control and GFP groups. However, at 24 h after delivery, the migration ability of CD151-ARSA mutant cells was reduced compared with the CD151 group, and the most notable effect was at 48 h (Fig. 3).

Thus, these results suggest that CD151 transfection promotes wound healing while the CD151-ARSA mutant transfection delays wound healing.

### Effects of CD151 and CD151-ARSA transfection on the capillary network formation of HUVECs

**Figure 4:**
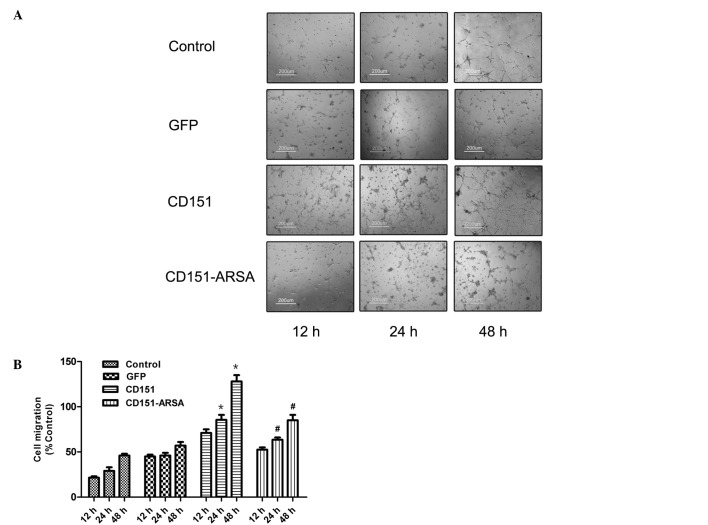
Effects of CD151 and CD151-ARSA transfection on the capillary network formation of HUVECs. (A) Representative photomicrographs observed at 12, 24 and 48 h after rAAV transfection on Matrigel showed that HUVECs assembled into capillary network structures. (B) Quantitative analysis of capillary network formation. ^*^p<0.05 vs. control and GFP group. ^#^p<0.05 vs. CD151 group. HUVECs, human umbilical vein endothelial cells; GFP, green fluorescent protein.

Upon plating on Matrigel basement membrane, HUVECs assembled into capillary network formation structures, which were observed at 12, 24 and 48 h after the rAAV transfection. As shown in Fig. 4A and B, CD151 transfection promoted the capillary network formation at 24 and 48 h after gene delivery, compared with the control and GFP groups (Fig. 4). By contrast, network formation was inhibited by transfection with the CD151-ARSA mutant at 24 and 48 h after gene delivery, compared with the CD151 group, and there was a marked difference at 48 h. These findings indicate that CD151-ARSA mutant delivery impairs the tube formation improved by CD151 gene transfer.

### Effects of CD151 and CD151-ARSA mutant on the PI3K/Akt and ERK signaling pathways

A number of signaling pathways are involved in angiogenesis, such as Akt, eNOS, ERK and p38 MAPK (26,27). We analyzed the levels of PI3K, Akt and ERK proteins, and applied inhibitors of MAPK (apigenin) and PI3K (LY294002) to HUVECs following transfection with CD151 and CD151-ARSA.

**Figure 5:**
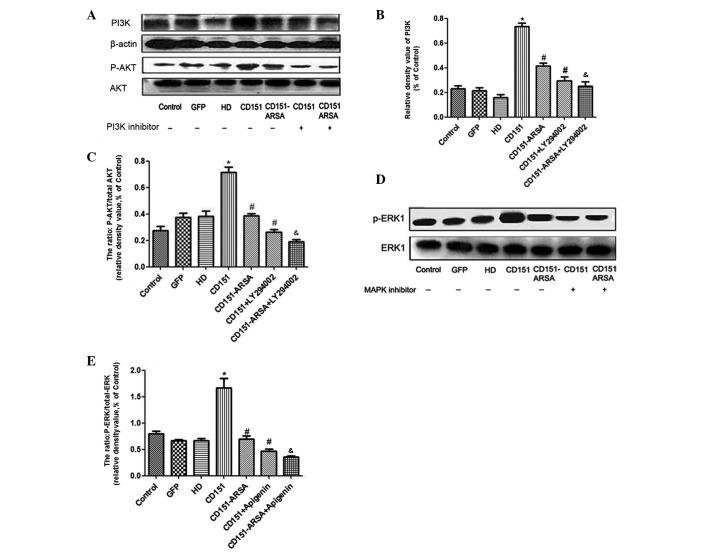
Effects of CD151 and CD151-ARSA transfection on PI3K/Akt and ERK signaling pathways. Western blot analysis for PI3K, phosphorylated Akt, total Akt, phosphorylated ERK and total ERK. Inhibitors of MAPK (apigenin) and PI3K (LY294002) were applied to HUVECs following transfection with CD151 or CD151-ARSA. (A-C) The protein levels of PI3K, Akt, phospho-Akt and quantitative analysis. (D and E) Levels of ERK1, phospho-ERK1 and quantitative analysis. Inhibitor of PI3K (LY294002, 15 μM); inhibitor of MAPK (apigenin, 25 μM). HD group (HD-Fugene 6 transfection reagent) was used as a control group. Each experiment was performed at least in triplicate. ^*^p<0.05 vs. control, GFP and HD groups. ^#^p<0.05 vs. CD151 group. ^&^p<0.05 vs. CD151-ARSA group (no signaling pathway inhibitor group). HUVECs, human umbilical vein endothelial cells; GFP, green fluorescent protein.

Consistent with earlier data, the present study showed the activation of PI3K/Akt and ERK signaling pathways in the CD151 group (15,16). However, in the CD151-ARSA group, the protein expression levels of PI3K, p-Akt and p-ERK were all reduced compared with the CD151 group (Fig. 5). The CD151-ARSA mutant resulted in reduced activation of PI3K/Akt and ERK signaling pathways (Fig. 5). In addition, MAPK inhibitor (apigenin) and PI3K inhibitor (LY294002) significantly reduced the activation of ERK and PI3K induced by CD151, respectively (Fig. 5).

As a whole, these results suggest that the gene transfer of CD151-ARSA mutant attenuates the activation of the PI3K/Akt and ERK signaling pathways.

## Discussion

The present study was designed to investigate the molecular mechanisms that govern the effects of CD151 in angiogenesis. In this *in vitro* study, our results showed that the delivery of the CD151-ARSA mutant (YRSL→ARSA) into HUVECs decreased cell migration and capillary network formation on Matrigel, contrary to the effects of CD151 gene delivery. Furthermore, we demonstrated that mutation of the CD151 YRSL motif resulted in diminished activation of PI3K/Akt and ERK signaling pathways in HUVECs. These data provide evidence that the CD151 YRSL motif was indeed a key region of CD151 for regulating cell migration, capillary network formation and angiogenesis.

Multiple lines of evidence indicate that the YRSL sequence of the CD151 C-terminal cytoplasmic tail is important and it is thought to determine its intracellular trafficking and function (24). These motifs in the cytoplasmic domain of CD151 could be recognized by adaptor protein (AP)-2 complex, a core component of clathrin endocytic machinery (28). It was found that the YRSL motif was required for CD151 endocytic processes, indicating that the YRSL sequence in the CD151 cytoplasmic domain determines its trafficking (22). When the YRSL→ARSA mutant human CD151 molecule was created and transfected into NIH3T3 mouse fibroblast cells, the vesicle trafficking was completely impaired and CD151-promoted NIH3T3 migration was diminished (22). Therefore, the YRSL sequence of CD151 is necessary for vesicle trafficking of CD151 and this sequence is also a key region for CD151-related cell migration.

Angiogenesis is a complex process involving extracellular matrix degradation, endothelial cell proliferation and migration, formation of tube structures and morphological differentiation (29–31). Although our previous data showed that CD151 was capable of promoting cell proliferation, cell migration and angiogenesis both *in vivo* and *in vitro*, the mechanisms remain to be elucidated. Based on above data, we hypothesized that the YRSL sequence of CD151 may also play an important role in the CD151-mediated process of angiogenesis. To test this hypothesis, in the present study we mutated the YRSL→ARSA motif in the human CD151 molecule and transfected it into HUVECs. As shown in the western blot analysis, CD151 and the CD151-ARSA mutant were both well expressed at the protein level, and the mutation did not influence the expression of CD151 protein. Furthermore, evidence indicates that the CD151-ARSA mutant transfection abrogates CD151-induced cell proliferation and migration *in vitro*. First, CD151-ARSA treatment decreased the HUVEC proliferation, in contrast to the promoting effects of CD151. Second, the directional motility of HUVECs (cell-wounding healing assay) showed that the CD151-ARSA mutant exhibited delayed cell motility, compared with CD151 transfectant. The YRSL motif was required for CD151 regulating cell migration, consistent with the results obtained from Liu *et al*(22).

Our previous work has shown that CD151 gene delivery promoted capillary formation *in vitro*(16). Although the specific region of CD151 QRD194–196 was emphasized (14,16), the YRSL motif may be another key region in CD151-induced angiogenesis. As was previously reported, endocytosis of adhesion molecules from cell-cell contacts may be regulated according to the YRSL motif (4,22,32). In particular, the capillary network formation was related to the maintenance of cell-cell contacts or junctions, which could be regulated by the lateral trafficking of cell adhesion molecules (4,21,22), and it was questioned whether CD151 may affect the capillary formation caused by the YRSL motif through vesicular trafficking. In the present study, it was shown that CD151-ARSA mutant transfection significantly disrupted the capillary network formation, while CD151 promoted the capillary network formation on Matrigel. The capillary network formation was inhibited by transfection with the CD151-ARSA mutant at 24 and 48 h after gene delivery. Notably, the difference became more significant over time, and a marked difference was observed at 48 h. Thus, it was observed that CD151 is capable of regulating the capillary network formation through the YRSL motif. Based on these data, it was accepted that the CD151 YRSL sequence is critical for CD151-mediated capillary network formation. Therefore, CD151 YRSL sequence-mediated vesicular trafficking may be another mechanism for CD151 regulation of angiogenesis *in vitro*.

The signaling mechanism of CD151 has been explored in the last decade (9,11,31). A growing list of signaling pathways that may be involved includes FAK, ERK and PI3K/Akt (11,13–15,31). Takeda *et al* showed that the adhesion-dependent activation of PKB/c-Akt and e-NOS was diminished in CD151-null mouse lung endothelial cells (11). In the present study, the CD151-ARSA mutant transfection resulted in diminished activation of PI3K/Akt and ERK signaling pathways, which was opposite to the findings with CD151 transfection. Thus, the activation of PI3K/Akt and ERK signaling pathways may be involved in CD151-mediated endocytosis trafficking.

In conclusion, our data demonstrated that the CD151-ARSA mutant abrogated cell proliferation, migration and capillary network formation in HUVECs. Furthermore, we observed the CD151-mediated activation of ERK and PI3K/Akt signaling pathways, but these effects were all impaired by CD151-ARSA gene delivery. Our observations emphasize that the specific YRSL region of CD151 is important for vesicle trafficking, which plays a key role in CD151-induced cell proliferation, migration and capillary network formation. Our study demonstrates for the first time the effect of CD151 YRSL in CD151-induced angiogenesis. Further studies should be performed and may provide indications for a better understanding of CD151 and CD151-ARSA via integrin-vesicle trafficking.
