# [Corrigendum] The tetraspanin CD151‑ARSA mutant inhibits angiogenesis via the YRSL sequence

**DOI:** 10.3892/mmr.2023.13069

**Published:** 2023-08-10

**Authors:** Dan Peng, Houjuan Zuo, Zhengxiang Liu, Jin Qin, Yuanlin Zhou, Pengcheng Li, Daowen Wang, Hesong Zeng, Xin A. Zhang

Mol Med Rep 7: 836–842, 2013; DOI: 10.3892/mmr.2012.1250

Subsequently to the publication of the above paper, an interested reader drew to the authors’ attention that, in Fig. 4A on p. 839, the ‘CD151/24 h’ and ‘CD151-ARSA/48 h’ panels appeared to contain overlapping sections of data, such that they were potentially derived from the same original source, where these panels were intended to show the results from differently performed experiments. The authors have re-examined their original data, and realize that the ‘CD151-ARSA/48 h’ panel was inadvertently placed incorrectly in the figure.

The revised version of Fig. 4, now containing the correct data for the ‘CD151-ARSA/48 h’ experiment in Fig. 4A, is shown below. Note that this error did not adversely affect either the results or the overall conclusions reported in this study. All the authors agree with the publication of this corrigendum, and are grateful to the Editor of *Molecular Medicine Reports* for allowing them the opportunity to publish this. They also wish to apologize to the readership of the Journal for any inconvenience caused.

## Figures and Tables

**Figure 4.**
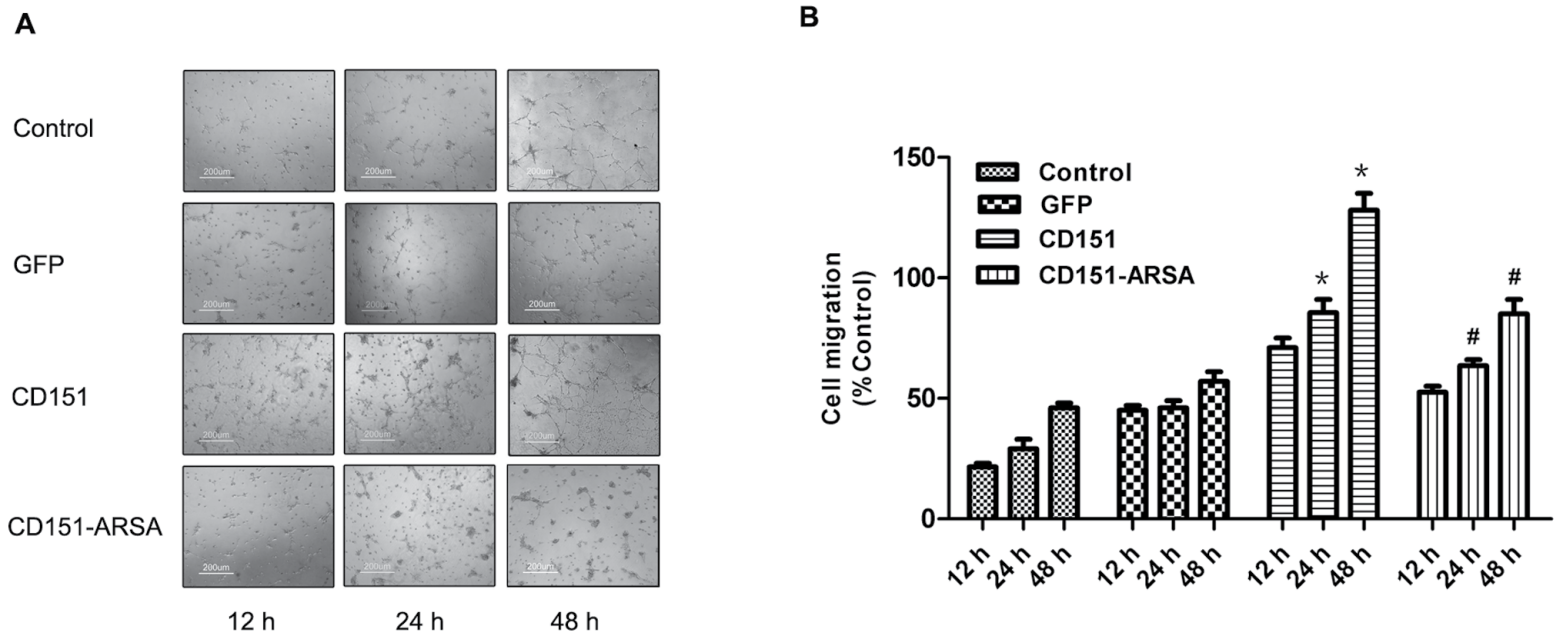
Effects of CD151 and CD151-ARSA transfection on the capillary network formation of HUVECs. (A) Representative photomicrographs observed at 12, 24 and 48 h after rAAV transfection on Matrigel showed that HUVECs assembled into capillary network structures. (B) Quantitative analysis of capillary network formation. *p<0.05 vs. control and GFP group. ^#^p<0.05 vs. CD151 group. HUVECs, human umbilical vein endothelial cells; GFP, green fluorescent protein.

